# Effect of Time Since Smoking Cessation on Lung Cancer Incidence: An Occupational Cohort With 27 Follow-Up Years

**DOI:** 10.3389/fonc.2022.817045

**Published:** 2022-03-01

**Authors:** Zheng Su, Xin-Hua Jia, Fang-Hui Zhao, Qing-Hua Zhou, Ya-Guang Fan, You-Lin Qiao

**Affiliations:** ^1^ Department of Epidemiology, National Cancer Center/National Clinical Research Center for Cancer/Cancer Hospital, Chinese Academy of Medical Sciences and Peking Union Medical College, Beijing, China; ^2^ The State Key Laboratory of Molecular Vaccinology and Molecular Diagnostics, National Institute of Diagnostics and Vaccine Development in Infectious Diseases, School of Public Health, Xiamen University, Xiamen, China; ^3^ Sichuan Lung Cancer Institute, Sichuan Lung Cancer Center, West China Hospital, Sichuan University, Chengdu, China; ^4^ Tianjin Key Laboratory of Lung Cancer Metastasis and Tumor Microenvironment, Tianjin Lung Cancer Institute, Tianjin Medical University General Hospital, Tianjin, China; ^5^ Center for Global Health, School of Population Medicine and Public Health Chinese Academy of Medical Sciences & Peking Union Medical College, Beijing, China

**Keywords:** smoking cessation, lung cancer, cohort, radon, arsenic

## Abstract

**Background:**

This special cohort reveals the effect of smoking cessation in occupational miners exposed to radon and arsenic.

**Methods:**

A total of 9,134 tin miners with at least 10 years of underground radon and arsenic exposure were enrolled beginning in 1992 and followed for up to 27 years. Detailed smoking information was collected at baseline, and information on smoking status was consecutively collected from 1992 to 1996. The Cox proportional hazards model was used to explore the relationship between time since smoking cessation and lung cancer.

**Results:**

A total of 1,324 lung cancer cases occurred in this cohort over 167,776 person-years of follow-up. Among populations exposed to radon and arsenic, miners after quitting smoking for 10 years or more had almost halved their lung cancer risk [adjusted hazard ratio (HR) = 0.55, 95% CI: 0.38–0.79], compared with current smokers. Among miners after quitting smoking for 5 years or more, lung cancer incidence approximately halved (HR = 0.52, 95% CI: 0.30–0.92) for squamous cell lung carcinoma, while it showed no significant decline for adenocarcinoma (HR = 0.79, 95% CI: 0.34–1.85).

**Conclusion:**

Smoking cessation for 10 years or more halved lung cancer incidence among miners exposed to radon and arsenic, and the benefit was more pronounced among squamous cell lung carcinoma.

## What This Paper Adds

What is already known about this subject?

Preliminary studies suggest that there exist joint effects between radon, arsenic, and smoking. Quitting smoking reduces not only smoking-related lung cancer, but also smoking-radon- and smoking-arsenic-related lung cancer. However, there is no prospective cohort to report the effect of years of smoking cessation on lung cancer incidence among miners exposed to radon and arsenic.

What are the new findings?

Among miners exposed to radon and arsenic, smoking cessation of at least 10 years would halve lung cancer incidence, and the benefit was more related to squamous cell lung carcinoma.

How might it impact policy in the foreseeable future?

To reduce the burden of lung cancer, smoking cessation is urgently needed among radon and arsenic miners. The longer years of smoking cessation should be emphasized among occupational miners than the general population.

## Background

Lung cancer remains the most common cancer with respect to both incidence and mortality, both in China and throughout the world (1, 2). Tobacco smoking is the leading risk factor for lung cancer, but other factors related to lung cancer include environmental tobacco smoke, air pollution, occupational exposures, marijuana, and other recreational drugs (3–6). Either radon or arsenic exposure is evident to be the major occupational carcinogens of lung cancer concluded by the International Agency for Research on Cancer (7, 8).

While it is clear that smoking cessation reduces lung cancer risk in general populations, this topic has been rarely investigated in occupational epidemiological studies. Population-based studies suggested a sharp decrease in lung cancer risk for over 50% in the first 5 years after smoking cessation (9, 10). However, findings from general population-based studies may not be directly generalized to occupational studies, because occupational workers additionally exposed to lung carcinogens generally have a higher lung cancer risk, and there exists a joint effect between cigarette use and occupational agents such as radon and arsenic. Consequently, the effect of smoking cessation on lung cancer tends to need a much longer time in occupational groups. A historical cohort of Chinese silicotics revealed that smoking cessation for 10 years halved lung cancer mortality among silicotics (11). Similarly, an asbestos-exposed cohort showed that lung cancer mortality rate ratio dropped steeply (over 50%) during the first 10 years after quitting smoking (12).

Globally, no studies to date have reported the effect of smoking cessation on lung cancer in occupational populations exposed to radon and arsenic. Among occupational radon cohorts, although Colorado Plateau cohort and German uranium miners collected individual smoking data, they have not revealed the effect of smoking cessation, and other cohorts generally lacked complete smoking information (13–15). Similarly, for occupational arsenic cohorts, most epidemiological studies of copper smelters in Utah, Sweden, Montana, and the United States have not reported the role of smoking cessation on lung cancer (16–19).

Among Chinese Tin miner studies, the results from case–control and cohort studies for several decades have identified that radon, arsenic, and smoking are the main risk factors for lung cancer (20–22). In addition, individual exposure information about radon, arsenic, and smoking was collected in our cohort. Therefore, it provided us a unique opportunity to investigate the effect of smoking cessation on lung cancer in workers exposed to radon and arsenic.

## Methods

### Study Design and Participants

**Figure 1:**
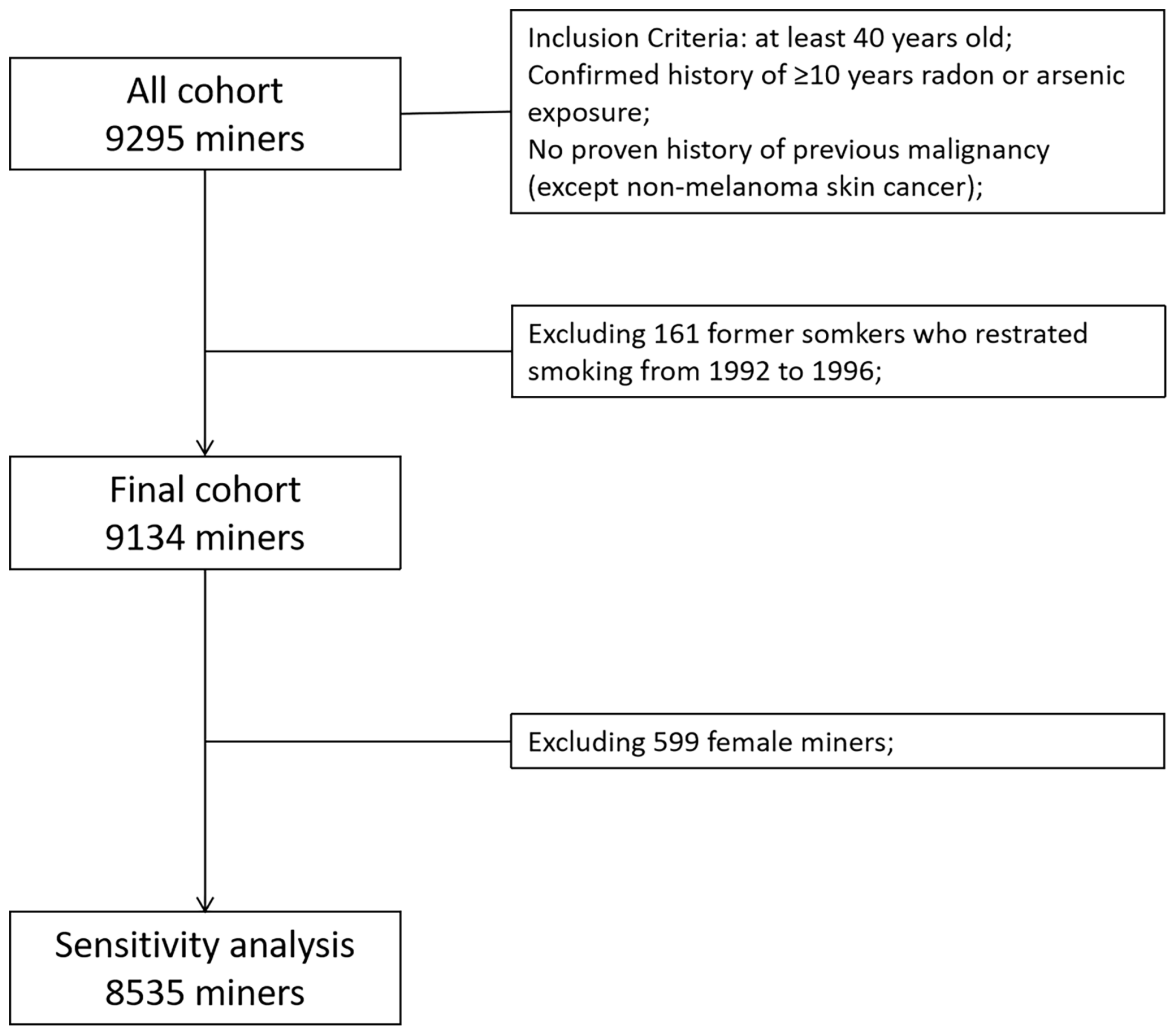
Flow chart of selections of the participants included in the statistical analysis.

The design and inclusion criteria of the Yunnan Tin Corporation (YTC) cohort was described previously (22, 23). Briefly, a total of 9,295 tin miners ≥ 40 years old who had 10 or more years of underground radon and/or arsenic exposure have been dynamically included into this cohort since 1992. All participants were followed by December 31, 2018. A total of 161 former smokers who restarted smoking from 1992 to 1996 were excluded. Then, 9,134 miners were included into the final analysis to estimate the risk of lung cancer incidence according to years since smoking cessation. In addition, as 599 women in this study were almost never smokers, all women were excluded. Finally, 8,535 male miners were included into a sensitivity analysis that was used to assess the robustness of analysis (**Figure 1**).

### Exposure Assessment

#### Tobacco Use

All miners were enrolled at baseline (in 1992) and information on age of start/stop smoking, type of tobacco (cigarette, waterpipe, and long-stem pipe), and smoking status was collected. According to the smoking status at baseline, we divided miners into smokers, former smokers, and never smokers. At baseline, individuals who had smoked regularly for 6 months or longer at any time in their lives were classified as smokers, and those who have a smoking duration of less than 6 months were considered never smokers and smokers who ceased smoking at enrollment were former smokers. In addition, to test the stability of smoking status, for each miner, information on smoking status and type of tobacco was collected for five consecutive years from 1992 to 1996. The change of smoking status for at least two consecutive years was identified as “the real behavior change” from 1992 to 1996. A total of 161 former smokers who restarted smoking were excluded.

However, the impact of type of cigarette was not considered in this study, because most of the participants were mixed smokers: at baseline, 62.4% (4,306/6,899) used cigarette + waterpipe, 29.3% (2,022/6,899) used cigarette only, and 8.3% (571/6,899) used waterpipe only among current smokers; 53.6% (1,084/2,022) of cigarette-only and 53.6% (306/571) of waterpipe-only smokers became mixed smokers in the next 4 years. Thus, the impact of type of cigarette was not considered in the final analysis.

Therefore, based on previous findings, we calculated a cigarette-equivalent variable adjusting conservatively: 1 g water pipe = 1 cigarette. Smoking intensity was measured by the number of cigarettes smoked per day and pack-years were calculated as the average number of cigarettes per day (divided by 20) times the number of years of smoking. Finally, smoking pack-years as a continuous variable was included into the multiple Cox proportional hazards model.

#### Occupational Carcinogens

Detailed definitions of occupational radon and arsenic exposures were given elsewhere (22, 23). In this study, according to total radon exposure, participants were classified into three groups: low group: <100 cumulative working level month (WLM), medium group: ≥100 and <400 WLM, and high group: ≥400 WLM. On the other hand, according to total arsenic exposure, participants were also classified into three groups: low group: <40 mg/m^3^, medium group: ≥40 and <100 mg/m^3^, and high group: ≥100 mg/m^3^. Finally, we combined these groups into three new groups named occupational exposure groups: lowly exposed group: low radon group and low arsenic groups; moderately exposed group: low radon–medium arsenic, medium radon–low arsenic, and medium radon–medium arsenic; highly exposed group: either high radon group or high arsenic group (a total of 5 subgroups: low radon–high arsenic, medium radon–high arsenic, high radon–high arsenic, high radon–low arsenic, and high radon–medium arsenic). In addition, other information including age, sex, education level, and prior lung disease was also collected for each participant.

#### Follow-Up and Case Ascertainment

From 1992 to 1999, annual follow-up was conducted combined with screening by chest radiography and sputum cytology. In 2005 and 2006, the first post-screening follow-up was performed and participants were followed until December 31, 2001. In 2019, an extended follow-up was conducted, and the survival status, total cause of death, cancer diagnosis, and death information were collected.

The primary outcome was lung cancer incidence, which was from the local cancer registration agency, medical record system, and funeral parlor, and face-to-face interviews with relatives and workmates of the participants. In the process of information extraction, participants’ name, age, work units, and home address were taken into consideration. By the end of December 31, 2018, 187 participants (2.1%) were lost to follow-up, with a follow-up rate of 97.9%.

Lung cancer cases were confirmed *via* the following ways: (1) histology (from surgical resection tissue or biopsy); (2) cytology (from sputum sample or endoscopy brushing); (3) x-ray; and (4) others (e.g., death certificate listing only without other information).

### Statistical Analysis

Person-years of follow-up were calculated from the date of enrollment to the date of lung cancer incidence or date of death or censoring as of December 31, 2018. Descriptive statistics was used to show various characteristics among never, former, and current smokers at the baseline. The association between time since smoking cessation and risk of lung cancer incidence was analyzed by the Cox proportional hazards model. Schoenfeld residuals were used to check the proportional hazards assumption. To control the effect of occupational exposures (radon and arsenic), Cox proportional hazards model was performed to calculate the hazard ratio (HR) and 95% confidence interval (95% CI) in three different occupational exposure subgroups (low, medium, and high groups). Furthermore, to reduce potential residual confounding by occupational exposure, we also modeled occupational exposure as continuous variables into the statistical analysis within each exposed stratum. In addition, in the multiple Cox proportional hazards model, other variables including age at entry, sex, education level, family history of lung cancer in first-degree relatives, and prior lung disease (silicosis, tuberculosis, asthma, and chronic bronchitis) were adjusted to eliminate confounding effects. Finally, since the loss of follow-up rates and missing data were very low, those with missing values were not included in the analysis. SAS and R software were used for statistical analysis.

## Results

The characteristics of the YTC miners in subgroups of smoking status at baseline are shown in **Table 1**. A total of 6,899 (75.5%) of them were current smokers, 772 (8.5%) were former smokers, and 1,463 (16.0%) were never smokers. The number of lung cancer cases and person-years were 1,075 and 124,797.3 in current smokers, 121 and 12,087 in former smokers, and 128 and 30,891.0 in never smokers, respectively. Almost all women (99.7%) were never smokers, and never smokers had the youngest age (mean = 49, IQR = 41–55) at enrollment. Smoking (number of pack-years in life time) in current smokers [mean = 26.4, interquartile range (IQR) = 14.2–34.5] was significantly higher than that in former smokers (mean = 18.7, IQR = 5.9–25.5). Compared with the non-smokers, current smokers had lower educational level, more prone to having prior lung disease (silicosis, tuberculosis, asthma, and chronic bronchitis), and higher occupational exposure.

**Table 1:** Characteristics of the YTC miners in subgroups of smoking status at baseline.

Characteristics	All subjects	Smoking status at baseline			*p*-value
		Current smokers	Former smokers	Never smokers	
**No. of subjects**	9,134	6,899 (75.5%)	772 (8.5%)	1,463 (16.0%)	
**Age (years)**					<0.01
Mean (IQR)	53 (43–61)	53 (43–61)	59 (53–66)	49 (41–55)	
**Gender**					<0.01
Male	8,535	6,897 (80.8%)	772 (9.1%)	866 (10.2%)	
Female	599	2 (0.3%)	0 (0.0%)	597 (99.7%)	
**Education**					<0.01
No	2,155	1,771 (82.2%)	223 (10.4%)	161 (7.4%)	
≤6 years	4,384	3,429 (78.2%)	354 (8.1%)	601 (13.7%)	
>6 years	2,595	1,699 (65.5%)	195 (7.5%)	701 (27.0%)	
**Family History of Lung Cancer**					<0.01
Yes	631	454 (71.0%)	40 (6.3%)	137 (21.7%)	
No	8,503	6,445 (75.8%)	732 (8.6%)	1,326 (15.6%)	
**Silicosis**					<0.01
Yes	444	349 (78.6%)	70 (15.8%)	25 (5.6%)	
No	8,690	6,550 (75.3%)	702 (8.1%)	1,438 (16.6%)	
**Tuberculosis**					<0.01
Yes	260	181 (69.6%)	40 (15.4%)	39 (15.0%)	
No	8,874	6,718 (75.7%)	732 (8.2%)	1,424 (16.1%)	
**Asthma**					<0.01
Yes	649	481 (74.1%)	119 (18.3%)	49 (7.6%)	
No	8,485	6,418 (75.6%)	653 (7.7%)	1,414 (16.7%)	
**Chronic bronchitis**					<0.01
Yes	2,363	1,856 (78.5%)	302 (12.8%)	205 (8.7%)	
No	6,771	5,043 (74.5%)	470 (6.9%)	1,258 (18.6%)	
**Pack-Years**					<0.01
Mean (IQR)	19.1 (7.8–31.0)	26.4 (14.2–34.5)	18.7 (5.9–25.5)	—	
**Occupational Exposure**					<0.01
Low group	1,143	690 (60.4%)	51 (4.5%)	402 (35.1%)	
Medium group	2,998	2,243 (74.8%)	201 (6.7%)	554 (18.5%)	
High group	4,993	3,966 (79.4%)	520 (10.4%)	507 (10.2%)	
**No. of Lung Cancer**	1,324	1,075 (81.2%)	121 (9.1%)	128 (9.7%)	
**No. of Person-Years**	167,776	124,797	12,314	30,891	

Values were given as n (%) for categorical variables, IQR (Q1Q3): interquartile range. p-value: the differences between the proportions were tested by Chi-square test, and the mean differences were tested by ANOVA between the subgroups. Mann–Whitney U test was carried out for the non-normal distribution data.

The evaluation of the stability of smoking status for 5 consecutive years in the YTC cohort is shown in **Table 2**. From current smokers to former smokers, there were only 1.6% (110/6,899); from never smokers to current smokers, there were only 2.1% (31/1,463). Among former smokers, the stability of smoking status varied significantly with increasing years since smoking cessation: the rates of quitting successfully were 57.6% (68/118) in those who quit smoking at enrollment, 74.0% (91/123) in 1 year after quitting, 77.2% (129/167) in 2–5 years after quitting, and 92.2% (484/525) in more than 5 years after cessation.

**Table 2:** Smoking status in five consecutive years from 1992 to 1996 in the YTC cohort.

The initial 4 years of follow-up				
	Smoking	Non-Smoking	Total	Relapse rate (%)^a^
**At baseline**				
Current smokers	6,789	110	6,899	1.6
Never smokers	31	1,432	1,463	2.1
**Years since cessation**			933	
<1	50	68	118	42.4
1	32	91	123	26.0
2-5	38	129	167	22.8
>5	41	484	525	7.8

a Relapse rate: During the initial 4 years of follow-up, smoking status for persistent change, that is, a change in status that remained for at least 2 years: current smokers quit, never smokers started smoking, and former smokers returned to smoking.

**Table 3** shows the effects of years of smoking cessation on lung cancer incidence among miners exposed to radon and arsenic. Generally, a significantly negative gradient (*p* < 0.001 for trend test) of lung cancer incidence was observed with increasing years of smoking cessation for all former smokers, despite the fact that significant risk reduction did not manifest within the first 1 year (HR = 1.03, 95% CI: 0.70–1.51), 2–5 years (HR = 0.85, 95% CI: 0.56–1.30), and 6–10 years (HR = 0.66, 95% CI: 0.43–1.03) of cessation. Furthermore, the risk of lung cancer incidence was nearly halved for 10+ years (HR = 0.55, 95% CI: 0.38–0.79). Furthermore, we observed the effect of smoking cessation stratified by radon and arsenic exposure. Among miners from the highly exposed group, they showed similar patterns to all miners, which is shown in **Table 3**. Given the low sample size in the lowly and moderately exposed group, data are shown in **Table S1** and the risk of lung cancer incidence among the lowly exposed group decreased by 50% (HR = 0.50, 95% CI: 0.24–1.16) within 5 years since cessation, and it further decreased by 65% (HR = 0.45, 95% CI: 0.11–1.89) if the smokers continued to abstain from cigarette smoking for 5 years or more. The beneficial effect was nearly similar in the moderately exposed group.

**Table 3:** Hazard ratio (HR, 95% confidence interval) of lung cancer incidence according to years of smoking cessation among the YTC miners.

Variable	No. of subjects	No. of lung cancer	No. of Person-Years	No. of lung cancer/Person-Years × 10^4^	Crude HR (95% CI)	Age-/Sex-Adjusted HR (95% CI)	Full-Adjusted HR (95% CI)^a^
**All miners without stratification**							
**Never Smokers**	1,463	128	30,869.7	41.5	0.46 (0.39, 0.56)	0.53 (0.42, 0.67)	0.67 (0.52, 0.85)
**Years since cessation**	772	100	12,078.9	82.8			
≤1	159	27	2,132.7	126.6	1.49 (1.02, 2.18)	0.98 (0.67, 1.44)	1.03 (0.70, 1.51)
2–5	152	22	2,337.1	94.1	1.08 (0.71, 1.65)	0.85 (0.56, 1.30)	0.85 (0.56, 1.30)
6–10	148	20	2,355.1	84.9	0.98 (0.63, 1.52)	0.68 (0.44, 1.06)	0.66 (0.43, 1.03)
>10	313	31	5,254.0	59.0	0.67 (0.47, 0.95)	0.48 (0.33, 0.68)	0.55 (0.38, 0.79)
**Current Smokers**	6,899	1,096	124,708.7	87.9	1	1	1
**Highly exposed group**							
**Never Smokers**	507	61	9,953.4	61.3	0.49 (0.38, 0.64)	0.57 (0.43, 0.76)	0.71 (0.52, 0.96)
**Years since cessation**	520	81	7,318.1	110.7			
≤1	97	20	1,104.9	181.0	1.55 (1.00, 2.42)	1.14 (0.73, 1.79)	1.10 (0.70, 1.71)
2–5	107	21	1,473.0	142.6	1.17 (0.76, 1.80)	0.97 (0.63, 1.50)	0.98 (0.63, 1.52)
6–10	103	17	1,478.7	115.0	0.95 (0.59, 1.53)	0.73 (0.45, 1.18)	0.70 (0.43, 1.14)
>10	213	23	3,261.6	70.5	0.58 (0.38, 0.87)	0.46 (0.30, 0.69)	0.53 (0.35, 0.80)
**Current Smokers**	3,966	803	65,478.1	122.6	1	1	1

a Multiple Cox proportional hazards models were adjusted for age, gender, education, family history of lung cancer, silicosis, tuberculosis, asthma, chronic bronchitis, radon, arsenic, and smoking pack-years. Lowly Exposed Group: low radon and low arsenic; Moderately Exposed Group: low radon–medium arsenic, medium radon–low arsenic, and medium radon–medium arsenic; Highly Exposed Group: high arsenic–low radon, high arsenic–medium radon, high radon–high arsenic, high radon–low arsenic, and high radon–medium arsenic. Radon exposure: low radon: <100 cumulative working level month (WLM), medium radon: ≥100 and <400 WLM, high radon: ≥400 WLM; Arsenic exposure: low arsenic: <40 mg/m^3^, medium arsenic: ≥40 and <100 mg/m^3^, high arsenic: ≥100 mg/m^3^.

As most women in our cohort were never smokers, a sensitivity test had been conducted and indicated that our analysis was robust (**Table S2**). We further analyzed the lung cancer incidence risk in relation to years of smoking cessation by histological type among the highly exposed group, as shown in **Table 4**. To reduce potential residual confounding by occupational exposure, we excluded miners in lowly and moderately exposed groups based on the quite low sample size of lung cancer cases. Results showed that for squamous cell carcinoma (SQC), the risks showed a significantly decreasing trend with increasing time since cessation (*p* < 0.001 for trend test), and finally, the risk in those quitting for over 5 years was 0.52 (95% CI: 0.30-0.92), which was similar to the risk in never smokers (HR = 0.56, 95% CI: 0.32–0.99).

**Table 4:** Hazard ratio (HR, 95% confidence interval) of lung cancer incidence according to years since smoking cessation by histologic types among highly exposed group.

Variable	No. of subjects	No. of lung cancer	No. of Person-Years	No. of lung cancer/Person-Years × 10^4^	Crude HR (95% CI)	Age-/Sex-Adjusted HR (95% CI)	Full-Adjusted HR (95% CI) ^a^
**Squamous Cell Carcinoma**							
**Never Smokers**	507	13	9,953.4	13.1	0.34 (0.20, 0.60)	0.51 (0.29, 0.89)	0.56 (0.32, 0.99)
**Years since cessation**	520	26	7,318.1	35.5			
≤5	204	13	2,577.8	50.4	1.24 (0.71, 2.16)	0.94 (0.54, 1.65)	0.92 (0.52, 1.61)
>5	316	13	4,740.3	27.4	0.69 (0.40, 1.21)	0.52 (0.30, 0.91)	0.52 (0.30, 0.92)
**Current Smokers**	3,966	257	65,478.1	39.2	1	1	1
**Adenocarcinoma**							
**Never Smokers**	507	5	9,953.4	5.0	0.37 (0.15, 0.91)	0.55 (0.22, 1.35)	0.72 (0.28, 1.89)
**Years since cessation**	520	11	7,318.1	15.0			
≤5	204	5	2,577.8	19.4	1.47 (0.60, 3.61)	1.12 (0.45, 2.77)	1.16 (0.47, 2.89)
>5	316	6	4,740.3	12.7	0.95 (0.42, 2.18)	0.71 (0.31, 1.62)	0.79 (0.34, 1.85)
**Current Smokers**	3,966	88	65,478.1	13.4	1	1	1

a Multiple Cox proportional hazards models were adjusted for age, gender, education, family history of lung cancer, silicosis, tuberculosis, asthma, chronic bronchitis, radon, arsenic, and smoking pack-years. Lowly Exposed Group: low radon and low arsenic; Moderately Exposed Group: low radon–medium arsenic, medium radon–low arsenic, and medium radon–medium arsenic; Highly Exposed Group: high arsenic–low radon, high arsenic–medium radon, high radon–high arsenic, high radon–low arsenic, and high radon–medium arsenic. Radon exposure: low radon: <100 cumulative working level month (WLM), medium radon: ≥100 and <400 WLM, high radon: ≥400 WLM; Arsenic exposure: low arsenic: <40 mg/m^3^, medium arsenic: ≥40 and <100 mg/m^3^, high arsenic: ≥100 mg/m^3^.

## Discussion

The YTC cohort with about 27 years of follow-up firstly reported that smoking cessation was associated with a substantial reduction in lung cancer incidence among underground miners exposed to radon and arsenic. For all lung cancer, about a 50% decrease in the risk of lung cancer incidence was shown in nearly 10 years for miners exposed to radon and arsenic. In addition, the long-term beneficial effect was weakened for adenocarcinoma, compared with squamous cell carcinoma.

In male participants among YTC miners, the risk of lung cancer among current smokers was lower than that of Western countries, Japan, Hong Kong, and the rest of mainland China (24–26). It could be due to the fact that the underground mines were relatively closed spaces and the high smoking rate of miners likely resulted in significant passive smoking exposure, even for non-smokers. Therefore, risk in non-smokers working underground in the mines was probably higher than that in non-smokers working in a less confined environment, and use of non-smokers with such exposure misclassification as a reference group would bias the relative risk estimates downward (toward the null). On the other hand, it may be that the effect of smoking is lower due to effect of these occupational exposures. Compelling evidence showed that there was a sub-multiplicative joint effect between occupational exposures (either radon or arsenic exposure) and smoking.

In addition, our results showed that the longer the smoking cessation time at baseline, the lower relapse rates for former smokers during the follow-up period. In cohort studies, smoking status at baseline might change over time, which resulted in bias in the true association between exposure (smoking status) and outcome (lung cancer risks). Therefore, a definition of smoking cessation with stable relapse rate was crucial in the cohort study, and our data showed that the relapse rate of quitting smoking at least 5+ years was as low as 7.8%. Because the definition of former smoker varied by study—quit smoking at least 6 months+ (27), 1+ years (28), 2+ years (10, 29, 30), or 5+ years (9)—more studies were needed to explore the optimal definition of former smokers.

Existing lines of evidence have illustrated a definitive benefit of smoking cessation in relation to lung cancer risks. Notably, smoking cessation is associated with a decrease in relative risk of lung cancer in former smokers compared to current smokers, but the absolute lung cancer risk in former smokers does not decrease from smoking cessation. However, the temporal pattern of this risk after smoking cessation is still controversial. In a previous case–control study in Hong Kong, Lap et al. observed that compared to current smokers, there is a rapid decrease in lung cancer risk across most histological types of lung cancer within the first 5 years of quitting, and then it almost remained constant. However, Sadik et al. had conducted a meta-analysis and found that a continued progressive reduction in lung cancer risk resulting from smoking cessation would remain at least 15 years (31). In addition, Paul et al. had conducted a pooled analysis and found that relative risk of lung cancer to the current smokers decreased gradually and continuously over years of smoking cessation (32). Similarly, data from the Prostate, Lung, Colorectal and Ovarian Cancer Screening Trial showed that in 30+ pack-year former smokers, smoking abstinence resulted in a gradual decrease in the risk of lung cancer death (33). Results from the National Lung Screening Trial also observed a steady decline in lung cancer death risk with the increase in duration of tobacco abstinence (34).

Generally, most epidemiological studies regarding smoking cessation and lung cancer risk were conducted in the general population, and it has been rarely investigated in occupational epidemiological studies. It appeared that this delayed decrease in lung cancer risk was more common among individuals with occupational exposure. A cohort study conducted among Australian workers exposed to asbestos found that the lung cancer mortality rate ratio among insulators dropped steeply during the first 10 years after quitting smoking (12). A large historical cohort of Chinese silicotics showed that the risk of lung cancer mortality among all silicotics was nearly halved within 20 years since cessation (adjusted HR = 0.54, 95% CI: 0.35–0.83) (11). It is well known that there exist joint effects between radon, arsenic, and smoking, and quitting smoking reduces not only smoking-related lung cancer, but also smoking-radon- and smoking-arsenic-related lung cancer (13, 35, 36). However, to our knowledge, there is no study reporting the effect of smoking cessation on radon- and arsenic exposed populations. Among the YTC miners, our results firstly showed that the benefits of smoking cessation were different in occupational groups exposed to radon and arsenic. For the lowly exposed group, a rapidly decreasing lung cancer incidence risk was shown within the first 5 years of smoking cessation, which was consistent with the moderately exposed group. However, it seemed to take longer years of smoking cessation to achieve the same reduction among the highly exposed group. Importantly, findings from lowly and moderately exposed groups should be viewed as tentative given the low sample size in these two groups, and more studies would be encouraged to focus on this field in the future.

In the YTC, the risk of SQC incidence among workers highly exposed to radon and arsenic was nearly halved after 15 years or more since cessation, but the reduction was smaller for ADC. The benefit of smoking cessation was more prominent for SQC, which was consistent with findings from other studies (10, 24, 27, 37). However, a case–control study in Chinese men showed that the relative risk for SQC decreased by 78% (95% CI: 22%–94%) after a smoker continued to abstain from cigarette smoking for 5 years or more (10). Therefore, it seems to be a delayed reduction of SQC among occupational populations highly exposed to radon and arsenic, compared to the general population.

The strength of this study was that it was a large, prospective population-based cohort that included detailed personal, occupational, and smoking information. There were still some limitations. The closed underground mines and the high smoking rate might have resulted in secondhand smoke exposure to non-smokers, and further biased risk estimates downward (toward the null). Moreover, the histology information was lacking for nearly half of lung cancer cases, which would decrease the statistical power when the analysis was conducted according to histology. Finally, 4.0% (53/1,324) of lung cancer cases was measured by the face-to-face interviews with relatives and workmates of the miners, which may be inaccurate and lead to recall bias. In this study, we had added the exposures (radon and arsenic) together without any weights, which might bias the results due to the different risks of lung cancer by radon and arsenic. Therefore, studies directly comparing the lung cancer risks from radon or arsenic should be conducted in the future.

In conclusion, our study firstly reported that among workers exposed to radon and arsenic, the benefit of smoking cessation is more related to squamous cell lung carcinoma. A tailored smoking cessation strategy is needed among the occupational population exposed to radon and arsenic.

## Data Availability Statement

The raw data supporting the conclusions of this article will be made available by the authors, without undue reservation.

## Ethics Statement

The studies involving human participants were reviewed and approved by the institutional review boards of the National Cancer Center/National Clinical Research Center for Cancer/Cancer Hospital, Chinese Academy of Medical Sciences. The patients/participants provided their written informed consent to participate in this study.

## Author Contributions

Y-GF and F-HZ had full access to all the data in the study and take responsibility for the integrity of the data and the accuracy of the data analysis. Conception and design: Y-LQ. Data collection: ZS and Y-GF. Analysis and interpretation: ZS. Drafting the article: ZS and X-HJ. Manuscript revision: ZS, Y-GF, and Y-LQ. All authors contributed to the article and approved the submitted version.

## Funding

This work was supported by Cancer Foundation of China (grant No. CFC2020KYXM001) and Key R & D projects of Science and Technology Department of Sichuan (grant No. 2020YFS0212). Tianjin Key Medical Discipline (Specialty) Construction Project (grant No. TJLCMS2021-02).

## Conflict of Interest

The authors declare that the research was conducted in the absence of any commercial or financial relationships that could be construed as a potential conflict of interest.

## Publisher’s Note

All claims expressed in this article are solely those of the authors and do not necessarily represent those of their affiliated organizations, or those of the publisher, the editors and the reviewers. Any product that may be evaluated in this article, or claim that may be made by its manufacturer, is not guaranteed or endorsed by the publisher.
