# Supplementary material for: Effect of Time Since Smoking Cessation on Lung Cancer Incidence: An Occupational Cohort With 27 Follow-Up Years
Source: Front Oncol. 2022 Mar 1;12:817045. doi: 10.3389/fonc.2022.817045 (PMC8921458; doi:10.3389/fonc.2022.817045)
Supplement: Supplementary file 1 [file Table_1.docx]

Table S1. Hazard ratio (HR, 95% confidence interval) of lung cancer incidence according to years of smoking cessation among the YTC miners among lowly and moderately exposed groups

| **Variable** | **No. of subjects** | **No. of lung cancer** | **No. of Person**  **-Years** | **No. of lung cancer/Person-Years** x **10^4^** | **Crude HR (95%CI)** | **Age-Sex-**  **Adjusted HR (95%CI)** | **Full-Adjusted HR (95%CI) ^a^** |
| --- | --- | --- | --- | --- | --- | --- | --- |
| Lowly Exposed Group | | | | | | | |
| **Never Smokers** | 402 | 27 | 9105.7 | 29.7 | 0.62 (0.40, 0.97) | 0.52 (0.26, 1.04) | 0.53 (0.24, 1.16) |
| **Years since cessation** | 51 | 4 | 1061.1 | 37.7 |  |  |  |
| <=5 | 23 | 2 | 466.1 | 42.9 | 0.92 (0.40, 3.75) | 0.69 (0.17, 2.85) | 0.50 (0.11, 2.18) |
| >5 | 28 | 2 | 595.0 | 33.6 | 0.73 (0.18, 2.97) | 0.62 (0.15, 2.55) | 0.45 (0.11, 1.89) |
| **Current Smokers** | 690 | 67 | 14466.8 | 45.6 | 1 | 1 | 1 |
| Moderately Exposed Group | | | | | | | |
| **Never Smokers** | 554 | 40 | 11810.6 | 33.9 | 0.67 (0.48, 0.93) | 0.51 (0.32, 0.81) | 0.64 (0.38, 1.08) |
| **Years since cessation** | 201 | 15 | 3699.8 | 40.5 |  |  |  |
| <=5 | 84 | 6 | 1425.9 | 42.1 | 0.85 (0.38, 1.91) | 0.61 (0.27, 1.38) | 0.57 (0.25, 1.30) |
| >5 | 117 | 9 | 2273.9 | 39.6 | 0.76 (0.39, 1.48) | 0.54 (0.28, 1.06) | 0.64 (0.32, 1.26) |
| **Current Smokers** | 2243 | 226 | 44763.8 | 50.5 | 1 | 1 | 1 |

^a^ Multiple Cox proportional hazards models were adjusted for age, gender, education, family history of lung cancer, silicosis, tuberculosis, asthma, chronic bronchitis, radon, arsenic and smoking pack-years. Lowly Exposed Group: low radon and low arsenic; Moderately Exposed Group: low radon-medium arsenic, medium radon-low arsenic and medium radon-medium arsenic; Highly Exposed Group: high arsenic-low radon, high arsenic-medium radon, high radon-high arsenic, high radon-low arsenic, high radon-medium arsenic. Radon exposure: low radon: <100 cumulative working level month (WLM) , medium radon: ≥100 and <400 WLM, high radon: ≥ 400 WLM; Arsenic exposure: low arsenic: <40 mg/m^3^, medium arsenic: ≥40 and <100 mg/m^3^, high arsenic: ≥ 100 mg/m^3^.

Table S2. Sensitivity analysis of lung cancer incidence according to years of smoking cessation among the YTC miners

| **Variable** | **No. of subjects** | **No. of lung cancer** | **No. of Person**  **-Years** | **No. of lung cancer/Person-Years** x **10^4^** | **Crude HR (95%CI)** | **Age-Sex-**  **Adjusted HR (95%CI)** | **Full-Adjusted HR (95%CI) ^a^** |
| --- | --- | --- | --- | --- | --- | --- | --- |
| All miners without stratification | | | | | | | |
| **Never Smokers** | 866 | 77 | 17671.6 | 43.6 | 0.49 (0.39, 0.61) | 0.53 (0.42, 0.67) | 0.67 (0.52, 0.85) |
| **Years since cessation** | 772 | 100 | 12078.9 | 82.8 |  |  |  |
| <=1 | 159 | 27 | 2132.7 | 126.6 | 1.49 (1.01, 2.18) | 0.98 (0.67, 1.43) | 1.02 (0.69, 1.49) |
| 2-5 | 152 | 22 | 2337.1 | 94.1 | 1.08 (0.71, 1.65) | 0.85 (0.56, 1.29) | 0.85 (0.56, 1.29) |
| 6-10 | 148 | 20 | 2355.1 | 84.9 | 0.98 (0.63, 1.52) | 0.67 (0.43, 1.05) | 0.66 (0.42, 1.03) |
| >10 | 313 | 31 | 5254 | 59 | 0.67 (0.47, 0.95) | 0.47 (0.33, 0.68) | 0.55 (0.38, 0.78) |
| **Current Smokers** | 6897 | 1096 | 124662.6 | 87.9 | 1 | 1 | 1 |
| Highly Exposed Group | | | | | | | |
| **Never Smokers** | 401 | 50 | 7649.6 | 65.4 | 0.52 (0.39, 0.70) | 0.58 (0.43, 0.77) | 0.71 (0.52, 0.96) |
| **Years since cessation** | 520 | 81 | 7318.2 | 110.7 |  |  |  |
| <=1 | 97 | 20 | 1104.9 | 181 | 1.54 (0.99, 2.40) | 1.14 (0.73, 1.78) | 1.09 (0.70, 1.70) |
| 2-5 | 107 | 21 | 1473 | 142.6 | 1.16 (0.76, 1.80) | 0.96 (0.62, 1.48) | 0.98 (0.63, 1.52) |
| 6-10 | 103 | 17 | 1478.7 | 115 | 0.94 (0.58, 1.53) | 0.72 (0.44, 1.16) | 0.70 (0.43, 1.13) |
| >10 | 213 | 23 | 3261.6 | 70.5 | 0.58 (0.38, 0.87) | 0.45 (0.30, 0.69) | 0.53 (0.35, 0.80) |
| **Current Smokers** | 3965 | 803 | 65455.5 | 122.7 | 1 | 1 | 1 |

^a^ Multiple Cox proportional hazards models were adjusted for age, gender, education, family history of lung cancer, silicosis, tuberculosis, asthma, chronic bronchitis, radon, arsenic and smoking pack-years. Lowly Exposed Group: low radon and low arsenic; Moderately Exposed Group: low radon-medium arsenic, medium radon-low arsenic and medium radon-medium arsenic; Highly Exposed Group: high arsenic-low radon, high arsenic-medium radon, high radon-high arsenic, high radon-low arsenic, high radon-medium arsenic. Radon exposure: low radon: <100 cumulative working level month (WLM) , medium radon: ≥100 and <400 WLM, high radon: ≥ 400 WLM; Arsenic exposure: low arsenic: <40 mg/m^3^, medium arsenic: ≥40 and <100 mg/m^3^, high arsenic: ≥ 100 mg/m^3^.
